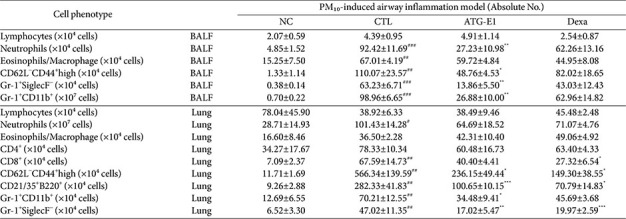# *Lacticaseibacillus paracasei* ATG-E1 Alleviates Particulate Matter-Induced Airway Inflammation in Association with Altered Inflammatory Signaling, Gut Microbiota, and Fecal Metabolites

**DOI:** 10.4014/jmb.2604.04029

**Published:** 2026-08-06

**Authors:** Hyunsoo Cho, Gunseok Park, Hyo-jeong Yun, Seungyeon Lee, Bokyeong Song, Miji Shin, Young-Sil Lee, Jihee Kang

**Affiliations:** Atogen, 11-6, Techno1-ro, Yuseong-gu, Daejeon, 34015, Republic of Korea

**Keywords:** Particulate matter, Airway inflammation, PM_10_D, *Lacticaseibacillus paracasei*, ATG-E1, Air pollution

## Abstract

Particulate matter (PM) acts as an environmental trigger for inflammatory airway diseases. This study investigated whether oral administration of *Lacticaseibacillus paracasei* ATG-E1 could therapeutically attenuate PM_10_ diesel exhaust particle (PM_10_D)-induced airway inflammation, and explored associated changes in inflammatory signaling, gut microbiota, and fecal metabolites. BALB/c mice were intranasally challenged with PM_10_D on days 0, 3, 6, and 8, and treated with ATG-E1 or dexamethasone after airway inflammation had been induced. Airway inflammation was evaluated using bronchoalveolar lavage fluid (BALF) cytology, flow cytometry, histopathology, enzyme-linked immunosorbent assay, reverse transcription quantitative polymerase chain reaction, and immunoblotting. Lung transcriptomics, cecal 16S rRNA profiling, and fecal metabolomics were performed, and antitussive and expectorant activities were assessed using ammonia-induced cough and phenol red secretion assays. ATG-E1 reduced inflammatory cell infiltration and neutrophilia in BALF, decreased collagen deposition, and lowered levels of pro-inflammatory mediators in BALF and lung tissue. ATG-E1 attenuated PM_10_D-activated IκBα and ERK phosphorylation, whereas JNK and p38 phosphorylation were not significantly altered by PM_10_D under the present experimental conditions. ATG-E1 also reduced caspase-1 and interleukin-1α expression. RNA sequencing revealed a broad downregulation of cytokine-cytokine receptor interaction signaling. ATG-E1 treatment was associated with gut microbiome remodeling, including enrichment of *Enterorhabdus* and *Butyricicoccus*, and altered fecal metabolite profiles were characterized by increased branched-chain fatty acids and decreased branched-chain amino acids. Functionally, ATG-E1 reduced cough frequency and increased tracheal phenol red output. Overall, *L. paracasei* ATG-E1 alleviated PM_10_D-induced airway inflammation and respiratory symptoms, in association with pulmonary immunomodulation and microbiome-associated fecal metabolite remodeling.

## Introduction

Air pollution is a major health risk worldwide. It is the second leading risk factor for premature death globally. According to the State of Global Air 2024 report [1], air pollution caused about 8.1 million deaths in 2021 [1-2]. Air pollution poses a particularly high risk in low- and middle-income countries in Southeast Asia and Africa due to elevated exposure levels [1]. Exposure to air pollution increases the risk of major diseases, including cardiovascular diseases (ischemic heart disease and stroke) [3], respiratory diseases (chronic obstructive pulmonary disease (COPD), asthma, lower respiratory infections), and lung cancer.

Particulate matter (PM) is a major component of outdoor and indoor air pollution and poses a threat to public health [4]. PM contains a complex mixture of solid and liquid particles dispersed in air with various chemical compositions. PM components typically include metals, inorganic ions, nitrates, sulfates, elemental and organic carbon, and polycyclic aromatic hydrocarbons [5]. The health impacts of PM are dependent on particle diameter. Finer particles can bypass the upper airway defenses and reach the alveoli, where they induce oxidative stress, disrupt immune regulation, and trigger inflammatory responses within pulmonary tissues [6]. Among the various components of PM, diesel exhaust particles (DEP) are one of the most hazardous components of urban air pollution. Airborne PM combined with DEP (PM_10_D) can deeply penetrate the lungs, compromise epithelial barrier integrity, and activate both innate and adaptive immune responses [7-8]. This leads to immune cell infiltration and increases the levels of inflammatory cytokines and various types of chemokines. Repeated exposure to PM_10_D is associated with reduced pulmonary function and airway inflammation. Therefore, novel therapeutic interventions that can safely and effectively modulate the associated pathological responses are required.

Probiotics help maintain intestinal homeostasis [9]. Additionally, they exert immunomodulatory effects in other mucosal environments, such as the respiratory tract [10-11]. Specific *Lactobacillus* strains can attenuate airway inflammation by modulating immune responses, downregulating pro-inflammatory signaling, and enhancing airway epithelial barrier function. Importantly, many of these protective effects in the respiratory system are thought to be mediated, at least in part, through communication between the gut microbiota and the lung immune system, often involving microbial metabolites [12-14]. These findings support the growing recognition of microbiome-based interventions as adjunct strategies with both therapeutic and disease-modifying potential in inflammatory airway diseases.

While previous studies have largely focused on the prophylactic effects of probiotics administered prior to PM exposure, the therapeutic potential of probiotics in treating established airway inflammation remains less understood. Building on our previous report showing that *Lacticaseibacillus paracasei* ATG-E1 attenuates PM_10_D-induced airway inflammation through immune regulation, the present study extends these findings in several important ways. First, we adopted a therapeutic regimen in which ATG-E1 administration was initiated after airway inflammation had been established, thereby directly evaluating its treatment potential rather than its prophylactic effects. Second, we integrated lung transcriptomics with targeted signaling analyses to examine PM_10_D-activated inflammatory signaling responses, including IκBα and ERK phosphorylation, and further assessed inflammasome-associated markers, such as caspase-1 and IL-1α, in lung tissue. Third, we coupled 16S rRNA profiling with targeted fecal metabolomics to characterize ATG-E1-associated changes in gut microbiota composition and fecal metabolite profiles, including branched-chain fatty acids and branched-chain amino acids. Finally, we assessed functional respiratory outcomes, including cough suppression and expectoration, to connect anti-inflammatory effects with symptom-relevant readouts. Together, these data provide an integrative multi-omics evaluation of ATG-E1 as a microbiome-associated therapeutic candidate for PM-induced airway inflammation.

## Materials and Methods

### Isolation of *Lactobacillus paracasei* ATG-E1

The strain *Lactobacillus paracasei* ATG-E1 was sourced from a neonatal fecal sample, which was voluntarily provided by an anonymous donor in Daejeon, Republic of Korea, in January 2016. For the isolation procedure, the fecal material was homogenized in a sterile bag with 0.85% (w/v) NaCl solution and subsequently subjected to serial dilution using the same saline solution. Aliquots of the dilutions were then spread onto De Man Rogosa Sharpe (MRS, Difco Lab., Inc., USA) agar plates and cultivated at 37°C for 24–48 h. Following incubation, individual colonies were selected for further analysis based on their morphological characteristics observed under a microscope and a negative result in the catalase test. A promising catalase-negative isolate was definitively identified using 16S rRNA gene sequencing. The resulting sequence was verified against the BLAST database, confirming its identity as *L. paracasei*, and the strain was designated ATG-E1. This isolate has been deposited in the Korean Collection for Type Cultures (KCTC 14245BP).

### Induction of PM_10_D-Induced Airway Inflammation in Mouse Model

Male BALB/c mice, aged 6–8 weeks, were obtained from Orient Bio Co., Ltd. (Republic of Korea). To induce respiratory inflammation, mice were challenged via intranasal administration of a particulate matter mixture on days 0, 3, 6, and 8. This mixture contained PM_10_ (150 μg/day, ERM CZ-120, Sigma-Aldrich, USA) and diesel exhaust particles (30 μg/day, DEP; SRM 2975, Sigma-Aldrich). From day 3, oral treatments for the ATG-E1(1 × 10^10^ CFU/mouse/day) and dexamethasone (3 mg/kg) groups were administered for 6 consecutive days. Dexamethasone was included as a well-established glucocorticoid anti-inflammatory drug and served as a broad anti-inflammatory positive control rather than a pathway-selective inhibitor [15]. On day 9, all mice were humanely euthanized, and samples of blood, bronchoalveolar lavage fluid (BALF), as well as lung and intestinal tissues, were harvested for subsequent analysis. All animal experiments were conducted under a protocol (No. DJUARB2019-021) reviewed and approved by the Committee for Animal Welfare at Daejeon University, in full compliance with the committee's guidelines.

### Evaluation of Expectorant Activity

To evaluate the expectorant effect, mice were orally administered either the vehicle, ATG-E1 (1 × 10^8^, 1 × 10^9^, or 1 × 10^10^ CFU/mouse) once daily for 14 days, or Levodropropizine (150 mg/kg) once daily for 5 days as a positive control. Following the final administration, a 5% phenol red solution was injected intraperitoneally. After 30 min, mice were euthanized, and the tracheas were excised. The tracheas were washed with 1 mL of saline, and the amount of secreted phenol red was determined by measuring the absorbance of the supernatant at 546 nm after the addition of 1N NaOH.

### Measurement of Antitussive Activity

For the antitussive activity assay, mice were orally administered either the vehicle, ATG-E1 (1 × 10^8^, 1 × 10^9^, or 1 × 10^10^ CFU/mouse) once daily for 14 days, or Theobromine (50 mg/kg) once daily for 5 days as a positive control. One h after the final administration, cough was induced by exposing the mice to a 13% ammonium hydroxide (NH_4_OH) solution for 1 min using a nebulizer in a closed chamber. The total number of coughs was then counted for the subsequent 6 min.

### Sample Preparation

*L. paracasei* ATG-E1 was incubated in MRS broth at 37°C for 16 h. The cells were obtained by centrifugation and washed three times with phosphate-buffered saline. The cell pellets were resuspended in a cryoprotectant solution and lyophilized using a freeze-dryer (FD8508, ilShinBioBase, Republic of Korea). The freeze-dried *L. paracasei* ATG-E1 powder was resuspended in saline and prepared daily for the *in vivo* experiments.

### BAL Fluid Analysis and Cytological Analysis

Following euthanasia, the lungs of the mice were lavaged with PBS to collect bronchoalveolar lavage (BAL) fluid. The BAL fluid was centrifuged at 1000 rpm for 5 min to pellet the cellular fraction. Harvested cells were washed and subsequently used for fluorescence-activated cell sorting (FACS). For cytological analysis, cells were cytospun onto glass slides at 1000 rpm for 4 min, fixed, and stained with Diff-Quick Stain (Baxter Healthcare Corp., USA). The number of neutrophils was then determined by microscopy.

### Digestion of Lung Tissue and Cell Isolation

Lung tissues were minced and incubated in PBS containing collagenase IV (1 mg/mL) and dispase (2 mg/mL) with shaking at 37°C. After incubation, the digested lung tissue was filtered through a cell strainer and centrifuged at 3000 rpm for 20 min. The total number of cells was counted using a hemocytometer.

### Flow Cytometry Analysis

Single cell suspensions were obtained from bronchoalveolar lavage fluid (BALF) and lung tissues were incubated with antibodies against CD3, CD4, CD8, CD69, CD62L, CD44, CD25, Gr-1, CD11b, CD11c, and B220. The cells were then washed with FACS buffer, fixed with 0.5% paraformaldehyde, and washed again. Analysis was performed on a FACSCalibur flow cytometer equipped with CellQuest software (BD Biosciences, USA).

### Reverse Transcription and Quantitative Real-Time PCR

Total RNA was extracted from the lung using TRIzol reagent (Thermo Fisher Scientific, USA), and 1 μg of total RNA was reverse-transcribed into cDNA using an AccuPower RT PreMix (Bioneer, Republic of Korea) according to the manufacturer's instructions. The qRT-PCR was performed on an Applied Biosystems 7500 Real-Time PCR System (Applied Biosystems, USA), following the manufacturer's instructions. The specific primer sets used were: *Cxcl1*, 5'-ccgaagtcatagccacac-3', 5'-gtgccatcagagcagtct-3'; *Mip2*, 5'-atgcctgaagaccctgccaa-3', 5'-ggtcagttagccttgcctttg-3'; *Tnfa*, 5'-ggctttccgaattcactggagcct-3', 5'-cccggcttcaaataaatacattcata-3'; *Nos2*, 5'-atccagagcttgaaggtgttg-3', 5'-ggctgtcagagcctcgtggctttgg-3'; *Tarc*, 5'-catccatctcgtgctacttgtgtt-3', 5'-catctatccagttggcctctgttt-3'; *Ptgs2*, 5'-gccgtcttgagcctggataac-3', 5'-tgtcacttggtcgaatttgttca-3'; *Trpv1*, 5'-catcttcaccacggctgcttac-3', 5'-cagacaggatctctccagtgac-3'; *Muc5ac*, 5'-agaatatctttcaggacccctgct-3', 5'-acaccagtgctgagcatactttt-3'; *Trpa1*, 5'-tgagatcgaccggagt-3', 5'-tgctgaaggcatcttg-3'; *Gapdh*, 5'-tgaagcaggcatctgaggg-3' and 5'-cgaaggtggaagagtgggag-3'. All samples were normalized to their corresponding expression of GAPDH, and the expression of the gene of interest in the treatment groups was compared to that of the CTL group.

### Immunoblot Analysis

Lung lysates were resolved by SDS-PAGE and transferred to Nitrocellulose (NC) membranes, and blocked with 5% bovine serum albumin in Tween-20 treated Tris buffered saline, followed by incubation with anti-p-P38, P38, p-JNK, JNK, p-ERK, ERK, p-IKB-α, IKB-α, and β-actin (Cell Signaling Technology, USA). The protein signal was visualized using the Image Analyzer, ChemiDoc Imaging system (BioRad, USA) after incubation with the ECL detection reagent (Cytiva, USA).

### Analysis of Inflammatory Mediators in BALF and Serum

The levels of CXCL-1, MIP-2, IL-17a, IL-1α, and TNF-α in BALF, as well as symmetric dimethyl-arginine in serum, were measured using ELISA kits according to the manufacturer's instructions (R&D Systems, USA).

### Histopathological Analysis of Lung Tissues

Lung tissues were fixed in 10% neutral-buffered formalin, dehydrated, embedded in paraffin, and sectioned at a thickness of 5 μm. The tissue sections were stained with either hematoxylin and eosin (H&E) or Masson’s trichrome.

### Immunofluorescence Staining and Quantification

Lung sections were deparaffinized and permeabilized with PBS containing 0.5% Nonidet P-40, and blocked with 2.5% horse serum and bovine serum albumin. The sections were incubated with anti-caspase-1 and anti-IL-1α antibodies at 4°C overnight, followed by incubation with fluorescein-conjugated secondary antibodies. Nuclei were counterstained with Hoechst 33342. Fluorescence signals were visualized using a fluorescence microscope (Nikon Instruments, Inc., Canada) under identical imaging conditions for all experimental groups and quantified using ImageJ (National Institute of Health, USA). Mean fluorescence intensity was measured after background subtraction and normalized to the analyzed tissue area. The same ROI selection criteria and ROI size were applied consistently to all immunofluorescence images. ROIs were selected from the peribronchial (airway-surrounding) region in all samples. A fixed ROI size of 198 × 198 pixels was used for every measurement. For each experimental group, three mice were analyzed. From each mouse, one tissue slide was examined, and three ROIs were selected per slide, resulting in nine ROIs per experimental group. Therefore, all quantitative analyses were performed using identical ROI selection criteria and ROI dimensions across all samples to ensure consistency and minimize bias.

### Short-Chain Fatty Acids Quantification by LC–MS/MS

Fecal short-chain fatty acids (SCFAs) were quantified using liquid chromatography-tandem mass spectrometry (LC–MS/MS). SCFA standards were obtained from Sigma-Aldrich. For metabolite extraction, fecal samples were suspended in methanol containing 1% (v/v) formic acid (Thermo Fisher Scientific) and stored at -20°C (200 mg/mL). The sample was then homogenized for 1 minute using a Vibra-Cell Processors VCX 750 (Sonics & Materials, Inc., USA), followed by vortexing for 10 minutes and incubation overnight at –20? After incubation, the samples were centrifuged at 14,000 rpm for 1 h at 4°C. The supernatants were filtered through polyvinylidene fluoride syringe filters with 0.22 μm pore size (Millipore, USA), and derivatized by mixing with 200 mM 3-nitrophenylhydrazine (3-NPH; Sigma-Aldrich) in 60% aqueous methanol and 120 mM N-(3-dimethylaminopropyl)-N'-ethylcarbodiimide (EDC; Sigma-Aldrich) in anhydrous methanol (2:1:1, v/v). The mixtures were vortexed for 2 min and incubated at 40°C for 1 h. LC–MS/MS analysis was conducted using an Exion LC system connected to a QTRAP 4500 mass spectrometer (AB SCIEX, USA). Chromatographic separation was achieved using a Unison UK-C18 HT column (100 × 2 mm, 3 μm particle size; Imtakt, USA) maintained at 40°C. The mobile phases consisted of water (Sigma-Aldrich) containing 0.1% (v/v) formic acid (A), and acetonitrile (Sigma-Aldrich) containing 0.1% (v/v) formic acid (B) at a flow rate of 0.35 mL/min. The injection volume was 1 μL, and the autosampler was set to 4°C. Gradient elution was performed as follows: 15% (B) for 2 min, linear increase from 15% to 40% (B) for 14 min, from 40% to 100% (B) for 2 min, returned to 15% (B) within 1 min and equilibration for 1 min. Multiple reaction-monitoring mode transitions were as follows: acetic acid, m/z 193.9 → 152.0 with declustering potential (DP) of -70 V, collision energy (CE) of -20 eV, and collision cell exit potential (CXP) of -9 V; butyric acid, m/z 221.9 → 151.9 with DP of -75 V, CE of -22 eV, and CXP of -11 V; propionic acid, m/z 207.9 → 165.1 with DP of -55 V, CE of -20 eV, and CXP of -9 V; isobutyric acid, m/z 221.6 → 179.0 with DP of -95 V, CE of -20 eV, and CXP of -9 V; and isovaleric acid, m/z 235.9 → 151.6 with DP of -75 V, CE of -24 eV, and CXP of -7 V. MS parameters were set as follows: ion spray voltage, 4,500 V; curtain gas, 25 psi; collision gas, medium; ion source gas 1, 50 psi; ion source gas 2, 50 psi; turbo spray temperature, 450?; entrance potential, -10 V; and negative ion mode. SCIEX OS 2.1.0 software (AB SCIEX) was utilized for data acquisition and processing, and further data analysis was carried out using Analyst 1.7.1 software (AB SCIEX).

### Cecal Microbiota Analysis

Genomic DNA was isolated from cecal contents using the PowerFecal DNA Isolation Kit (Mo Bio Laboratories, USA). DNA concentration was quantified with a Qubit Flex Fluorometer (Thermo Fisher Scientific), and its integrity was assessed using the LabChip GX Touch HT platform (PerkinElmer, USA). The bacterial 16S rRNA gene, specifically the V3–V4 hypervariable regions, was PCR-amplified with unique 8-bp barcodes. Amplicons were sequenced on the Illumina MiSeq PE300 system following the appropriate protocol [16]. All raw sequencing data have been deposited in the NCBI SRA database under BioProject accession PRJNA1009041. Data processing was carried out with the Quantitative Insights Into Microbial Ecology pipeline [17], including quality filtering of reads, and clustering into operational taxonomic units at 97% similarity against the SILVA 138.1 reference database [18]. Sequencing quality metrics, including total read counts and observed richness values for each sample, are provided in Supplementary Table S1. One ATG-E1-treated sample, LH8, yielded only 4,576 reads and was excluded from 16S rRNA-based microbiome analyses because its low sequencing depth could underestimate observed richness and would require excessive rarefaction of the remaining samples. After excluding LH8, alpha- and beta-diversity analyses were performed at a rarefaction depth of 12,000 reads per sample. Rarefaction curve analysis was additionally performed to evaluate sequencing depth adequacy and coverage saturation across samples (Supplementary Fig. S5). Taxonomic classification was performed from phylum through genus levels. Both unweighted and weighted UniFrac distance matrices were calculated and visualized using principal coordinate analysis [19]. Differentially abundant taxa between groups were identified using the Linear discriminant analysis Effect Size (LEfSe) method, with statistical significance defined as Linear discriminant analysis (LDA) score > 2.0 and false discovery rate-adjusted *p* < 0.1.

### Transcriptome Analysis

Lung samples from each experimental group were stabilized and preserved in RNAlater solution (Invitrogen, USA) and sent to Macrogen Inc. (Republic of Korea) for library preparation (TruSeq Standed Total RNA LT Sample Prep Kit (Gold), Illumina, USA). RNA Sequencing (100 bp, NovaSeq 6000 S4 Reagent Kit, Illumina) was performed to produce raw data [20]. The resulting RNA sequencing data were quality checked using FastQC v. 0.11.7 and trimmed for Illumina adapters using Trimmomatic v. 0.38 [21]. All sequences were mapped using HISAT2 v. 2.1.0 [22], and mm10 was used as the reference genome. StringTie program v. 2.1.3b [22] was used to assemble known genes/transcripts using the reference gene model. Normalization of each sample was performed using the DESeq2 package in R, and fold change and p-values were calculated for each comparison [23]. Based on the results of the DEG analysis, GSEA (Gene Set Enrichment Analysis) was performed for each comparison group using the clusterProfiler package in R [24]. GSEA was conducted using the KEGG (Kyoto Encyclopedia of Genes and Genomes) database to identify pathways associated with specific gene sets and to calculate *p*-values for each pathway to select statistically significant ones [25]. KEGG mapper was used to confirm the expression levels of genes within the pathways (https://www.genome.jp/kegg/mapper/color.html). The raw sequencing data produced in the present study were deposited in NCBI's SRA database (NCBI BioProject PRJNA1009041).

### Statistical Analysis

All data are expressed as the mean ± standard error of the mean (SEM). Statistical analysis was performed using a one-way analysis of variance followed by Dunnett's multiple comparison test with GraphPad Prism 8.0 (GraphPad Software, USA). A *p*-value < 0.05 was considered statistically significant.

## Results

### Attenuation of Airway Inflammation and Fibrosis by *L. paracasei* ATG-E1

**Fig. 1:**
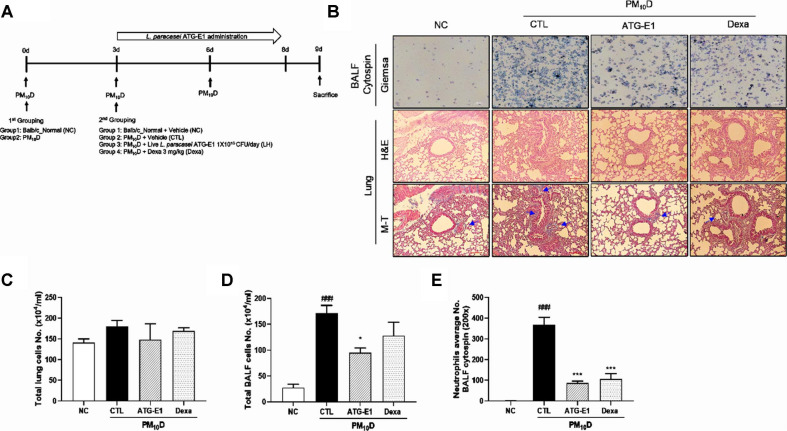
*L. paracasei* ATG-E1 ameliorated PM_10_D-induced airway inflammation in a mouse model. (**A**) Experimental schematic. (**B**) Representative images of Giemsa-stained bronchoalveolar lavage fluid (BALF) cells, hematoxylin and eosin (H&E)-stained lung tissue, and Masson's trichrome-stained lung tissue (collagen in blue). (**C**) Total cells in lung homogenates. (**D**) Total inflammatory cells in BALF. (**E**) Neutrophil counts in BALF. Data are presented as the mean ± standard error of the mean (SEM) (n = 5-8). ^###^*p* < 0.001, ^####^*p* < 0.0001 compared to the Normal Control (NC) group. ***p < 0.001 compared to the PM_10_D-treated control (CTL) group.

To investigate the therapeutic effects of *L. paracasei* ATG-E1 on airway inflammation, we treated mice with ATG-E1 following exposure to PM_10_D. Our experimental design specifically assessed the capacity of ATG-E1 to attenuate established inflammatory effects in the lungs and associated immune compartments (Fig. 1A). These effects were indicated by a marked reduction in inflammatory cell infiltration in BALF, as well as by histological improvements in lung tissue. H&E-stained lung tissues in the ATG-E1 group showed decreased peribronchial and perivascular inflammation compared with those in the control group. Furthermore, Masson’s Trichome staining revealed a marked reduction in collagen deposition, indicating the attenuation of airway fibrosis (Fig. 1B). Consistent with the histological improvements, quantitative analysis of BALF demonstrated a significant reduction in total inflammatory cell and neutrophilic infiltration levels (Fig. 1C–1E), demonstrating the therapeutic efficacy of ATG-E1.

### Immunomodulatory Effects of *L. paracasei* ATG-E1 on BALF and Lung Immune Cell Populations

Next, we examined the immunological alterations induced by ATG-E1 treatment in a PM_10_D exposed airway inflammation model. Immune cell populations in BALF and lung tissues were quantified (Table 1). PM_10_D induced a significant increase in the abundance of neutrophils (Gr-1+SiglecF-), eosinophils/macrophages, effector memory T cells (CD62L^−^CD44^+high^), and myeloid derived suppressor cells (Gr-1^+^CD11b^+^) in both the BALF and lung tissues compared with those in the NC group. Moreover, PM_10_D exposure significantly increased the abundance of tissue-resident memory B cells (CD21/35^+^B220^+^) and cytotoxic T cells (CD8^+^) in lung tissues. Treatment with ATG-E1 significantly reduced the number of neutrophils (Gr-1^+^SiglecF^−^), effector memory T cells (CD62L^-^CD44^+^high), and myeloid derived suppressor cells (Gr-1^+^CD11b^+^) cells in the BALF as well as that of tissue-resident memory B cells (CD21/35^+^B220^+^) in lung tissues. ATG-E1 treatment significantly lowered the neutrophil count in the BALF to a similar extent as dexamethasone. In contrast, there were no significant differences in eosinophil, macrophage, or lymphocyte counts after treatment with ATG-E1. Notably, the lung tissues of ATG-E1-treated mice exhibited lower levels of cytotoxic T cells (CD8^+^) than those of the control group, suggesting modulation of T cell-mediated immune responses by ATG-E1.

### Modulation of Pro-Inflammatory Mediators by *L. paracasei* ATG-E1

**Fig. 2:**
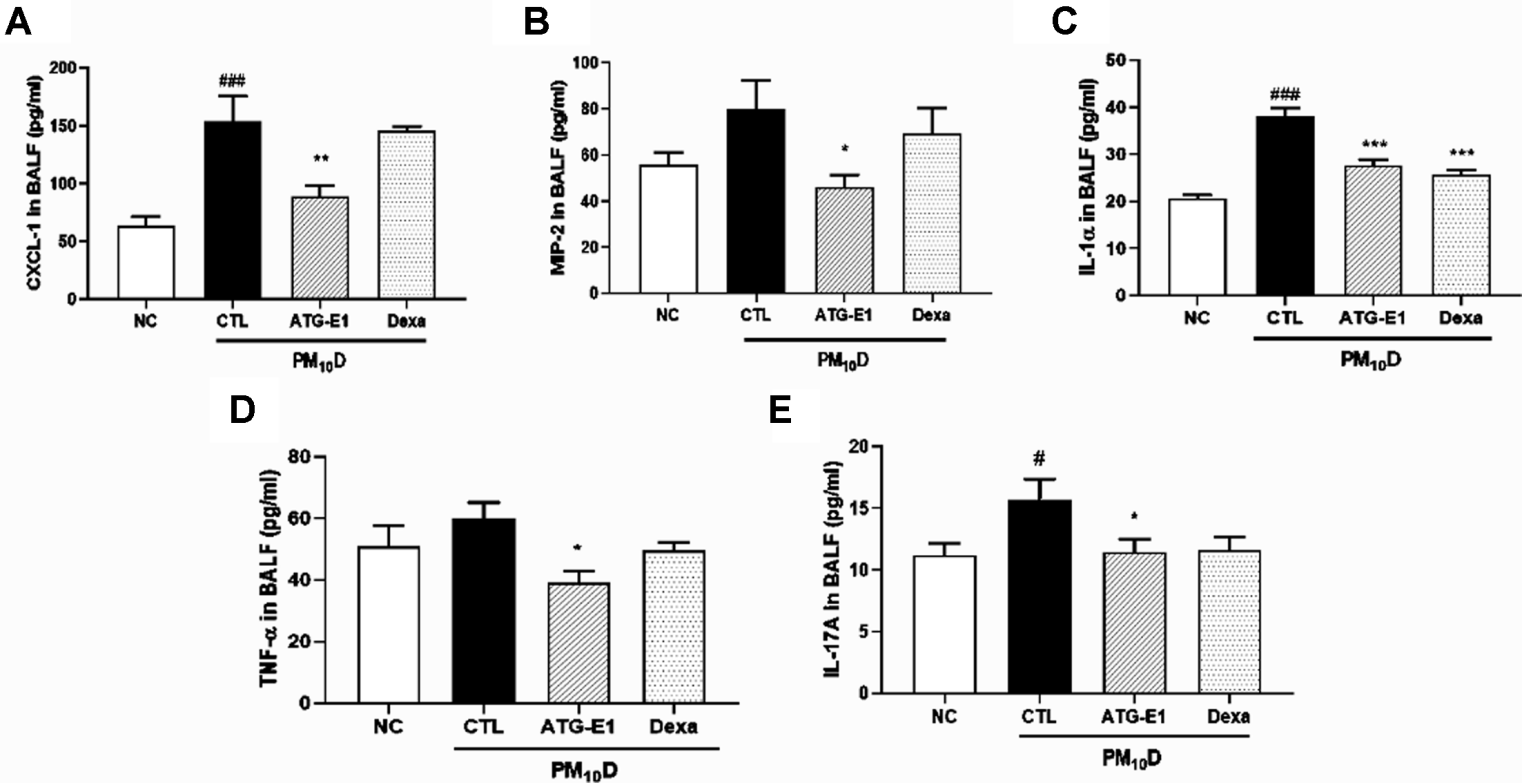
*L. paracasei* ATG-E1 treatment attenuates the expression of pro-inflammatory mediators in the airways of PM_10_D-exposed mice. Concentrations of key pro-inflammatory mediators in the bronchoalveolar lavage fluid (BALF) were measured. Levels of the neutrophil-attracting chemokines (**A**) CXCL-1 and (**B**) MIP-2, and the pro-inflammatory cytokines (**C**) IL-1α, (**D**) TNF-α, and (**E**) IL-17 are shown. Data are shown as the mean ± standard error of the mean (SEM). ^#^*p* < 0.05, ^###^*p* < 0.001 vs. NC group; *p < 0.05, **p < 0.01, ***p < 0.001 vs. control (CTL) group.

Next, we investigated the effect of ATG-E1 on the levels of the airway inflammatory mediators CXCL-1, MIP-2, IL-1α, TNF-α, and IL-17A in the BALF (Fig. 2A–2E). Exposure to PM_10_D significantly increased the cytokine levels compared with those in the sham group. Administration of ATG-E1 following PM_10_D exposure significantly decreased cytokine levels. The levels of TNF-α, CXCL-1, and MIP-2 in the ATG-E1 group tended to be lower than those in the dexamethasone-treated group. In contrast, the reductions in IL-1α and IL-17A expression in the dexamethasone and ATG-E1 groups were comparable. These results suggest that ATG-E1 exerts anti-inflammatory effects in the BALF, similar to that of dexamethasone, with potential differences depending on the cytokine type.

### Global Transcriptional Downregulation of Inflammatory Pathways in Lung Tissues

To explain the molecular changes in lung tissue, transcriptome-based DEG analysis followed by GSEA revealed that the cytokine-cytokine receptor interaction pathway was significantly enriched, with a negative normal enrichment score, indicating that genes in this pathway were generally downregulated in the ATG-E1 group compared to the CTL group (Supplementary Fig. 1). Consistently, KEGG pathway mapping showed that ATG-E1 treatment suppressed the major immune pathways responsible for driving inflammatory responses (Supplementary Fig. S2). Specifically, this suppression included the downregulation of key genes involved in immune cell trafficking and activation, such as crucial chemokines (*CCL6*, *CXCL1*, *CXCL4*, and *CXCl7*) and their receptors (*CCR3*, *CCR7*, *CXCR4*, and *CXCR7*), interleukins and their receptors (*IL-5RA*, *IL-16*, and *IL-18*), and members of the TNF (Tumor Necrosis Factor) receptor superfamily, such as *TRAIL*, *CD40*, and *TROY*.

### Modulation of Airway Inflammatory and Irritation-Related Genes by *L. paracasei* ATG-E1

**Fig. 3:**
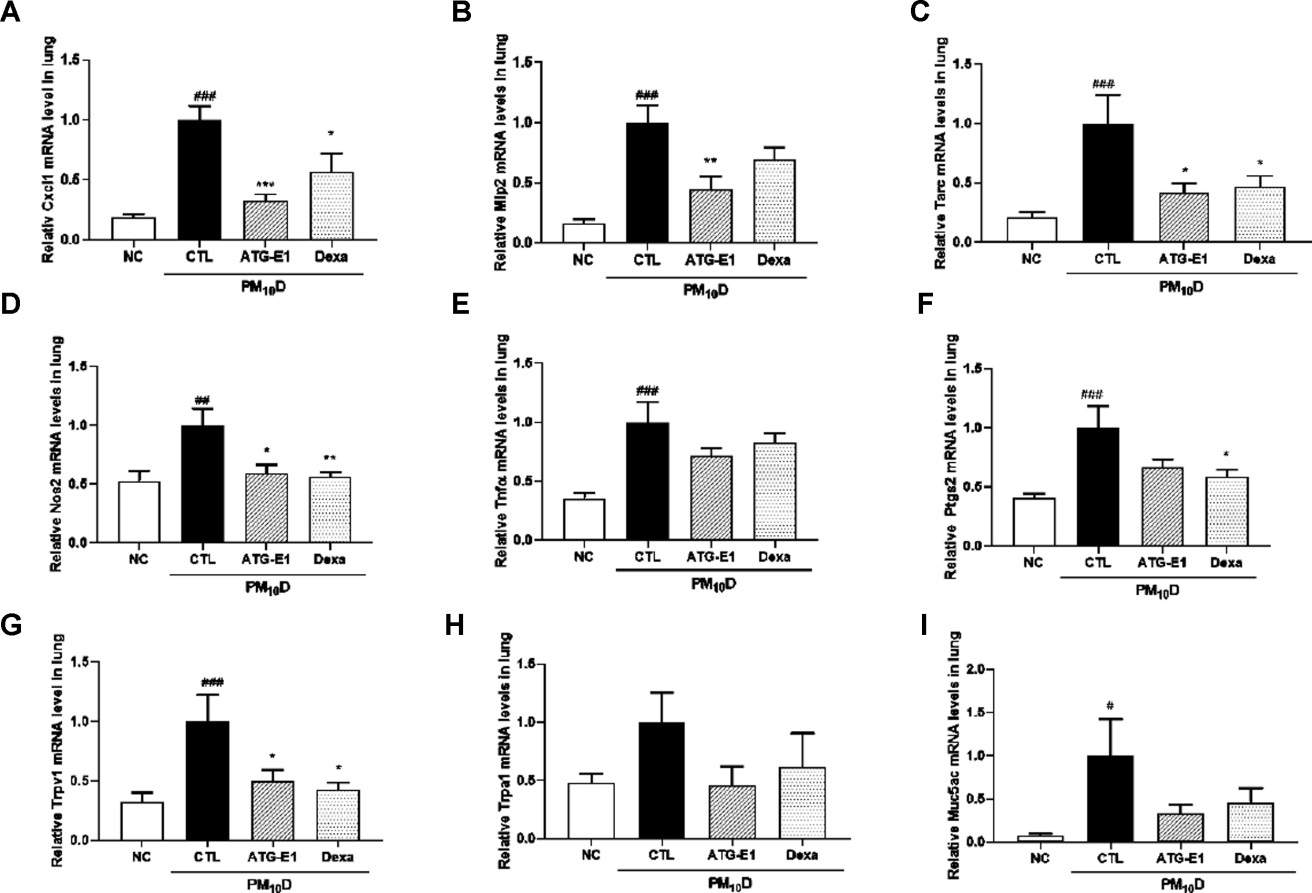
Attenuation of PM_10_D-induced inflammatory gene expression in the lungs by *L. paracasei* ATG-E1. Relative mRNA expression of pro-inflammatory chemokines (**A−C**) and other inflammatory mediators (**D−F**) was quantified in lung tissues by RT-qPCR. (**A**) *Cxcl1*, (**B**) *Mip2*, (**C**) *Tarc*, (**D**) *Nos2*, (**E**) Tnfα, (**F**) *Ptgs2*, (**G**) *Trpv1*, (**H**) *Trpa1*, and (**I**) *Muc5ac*. ATG-E1 administration markedly reduced the expression of these genes compared to the PM_10_D-treated control group. Results are expressed as the mean ± standard error of the mean (SEM). Statistical significance is denoted as ^##^*p* < 0.01, ^###^*p* < 0.001 compared to the normal control (NC) group; *p < 0.05, **p < 0.01, ***p < 0.001 compared to the PM_10_D-treated control (CTL) group.

To validate and further investigate the anti-inflammatory effects of ATG-E1 at the transcriptional level, we assessed the mRNA expression of various mediators in the lung tissues of mice. PM_10_D exposure significantly increased the mRNA expression of pro-inflammatory chemokines and cytokines, including *Cxcl1*, *Mip2*, *Tarc*, and *Tnfα* in the CTL group compared with that of the NC group (Fig. 3). The expression of genes associated with inflammation, such as *Nos2* and *Ptgs2*, was also significantly elevated following PM_10_D exposure. Furthermore, PM_10_D exposure upregulated the expression of genes related to sensory irritation and mucus production, namely *Trpv1* and *Muc5ac*. Treatment with ATG-E1 effectively counteracted these PM_10_D-induced changes. Specifically, ATG-E1 administration significantly decreased the mRNA levels of *Cxcl1*, *Mip2*, *Tarc*, *Nos2*, and *Trpv1* (Fig. 3A−3D, 3G). Although not statistically significant, the expression of *Tnfα*, *Ptgs2*, *Trpa1*, and *Muc5ac* also showed a clear decreasing trend in the ATG-E1 group compared with that in the CTL group (Fig. 3E, F, H, I). These results indicate that ATG-E1 mitigates PM_10_D-induced airway inflammation by downregulating a wide variety of genes related to inflammation, sensory irritation, and mucus production.

### *L. paracasei* ATG-E1 Attenuates PM_10_D-Activated IκBα and ERK Phosphorylation

**Fig. 4:**
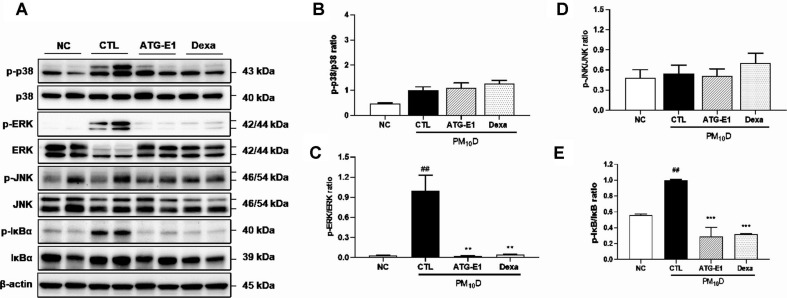
*L. paracasei* ATG-E1 attenuates PM_10_D-activated IκBα and ERK phosphorylation in lung tissues. (**A**) Representative western blot images for MAPK and NF-κB pathway proteins in lung tissue. Densitometric analysis of the relative protein expression of (**B**) p-p38/p38, (**C**) p-ERK/ERK, (**D**) p-JNK/JNK, and (**E**) p-IκBα/ IκBα. Data are presented as the mean ± standard error of the mean (SEM). ^##^*p* < 0.01, ^###^*p* < 0.001 compared to the normal control (NC) group. *p < 0.05, **p < 0.01, ***p < 0.001 compared to the PM_10_D-treated control (CTL) group.

Next, we investigated the effect of ATG-E1 on NF-κB and MAPK signaling using western blotting. PM_10_D exposure significantly increased the p-IκBα/IκBα and p-ERK/ERK ratios compared with the sham group, whereas p-JNK/JNK and p-p38/p38 ratios were not significantly altered in the quantitative analysis (Fig. 4A–4E). ATG-E1 treatment significantly reduced PM_10_D-induced IκBα and ERK phosphorylation to levels comparable to those observed in the sham and dexamethasone groups (Fig. 4C and 4E). These results suggest that ATG-E1 attenuated the NF-κB and ERK related signaling responses activated by PM_10_D under the present experimental conditions.

### *L. paracasei* ATG-E1 Attenuates the Expression of Canonical Inflammasome Activation Markers in Lung Tissues

**Fig. 5:**
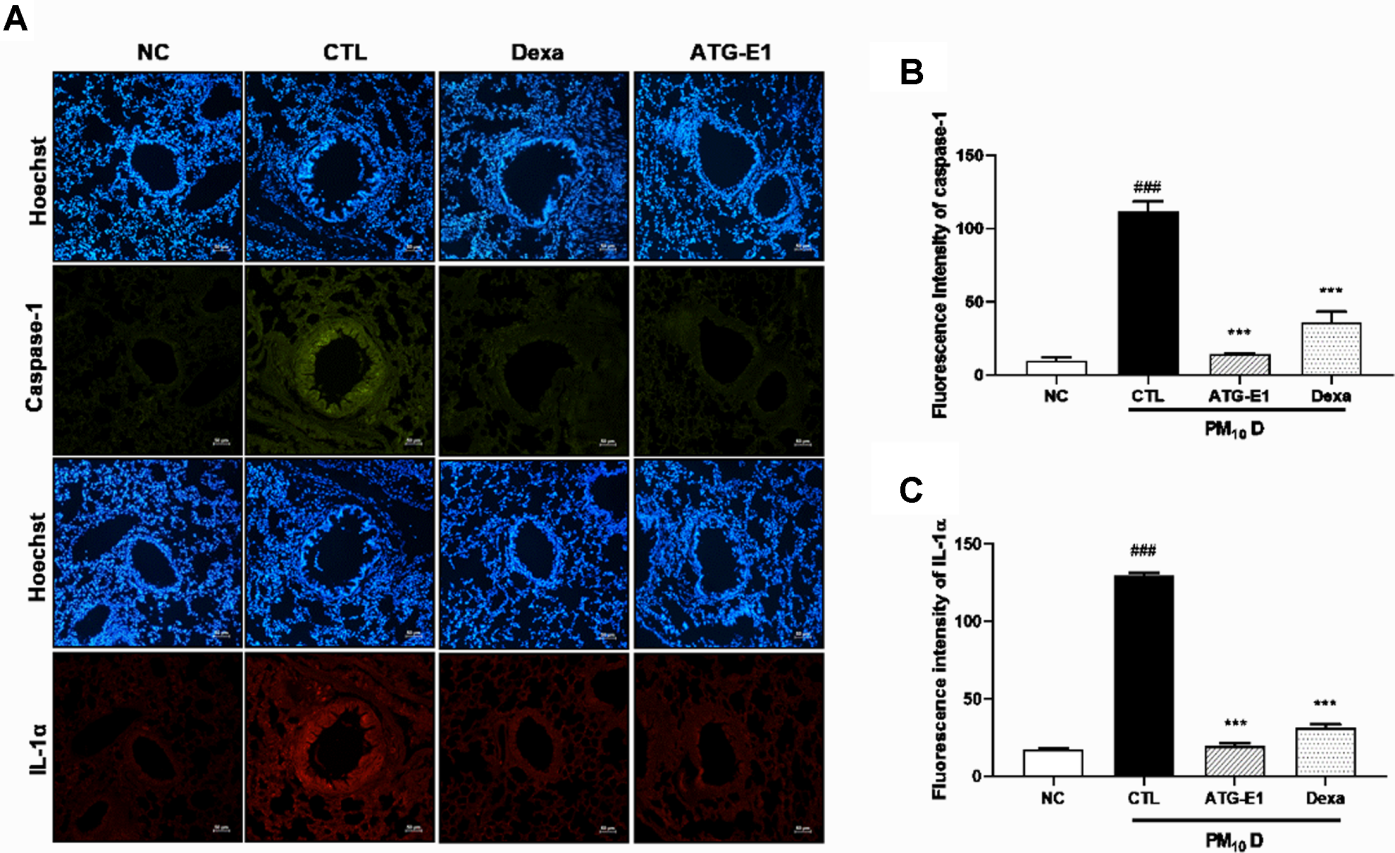
*L. paracasei* ATG-E1 protects against PM_10_D-Induced upregulation of pyroptotic markers in the lungs. (**A**) Representative immunofluorescence images of lung tissue sections stained for Caspase-1 (green), IL-1α (red), and nuclei (Hoechst, blue). Scale bar = 20 μm. (**B, C**) Quantification of the fluorescence intensity of (**B**) Caspase-1 and (**C**) IL-1α from the tissue sections. Data are presented as the mean ± standard error of the mean (SEM). ^###^*p* < 0.001 versus the normal control (NC) group; ***p < 0.001 versus the control (CTL) group.

Given that PM exposure can trigger inflammatory cell death pathways, we examined the effect of ATG-E1 on key proteins associated with pyroptosis by assessing the expression of caspase-1 and IL-1α in lung tissues. Immunofluorescence analysis showed that PM_10_D exposure significantly increased the expression of both proteins compared with that in the sham group (Fig. 5). Notably, treatment with ATG-E1 significantly suppressed PM_10_D-induced upregulation of caspase-1 and IL-1α with an inhibitory effect comparable to that of the positive control dexamethasone.

### *L. paracasei* ATG-E1 Treatment Was associated with Gut Microbiota Remodeling in PM_10_D-Exposed Mice

**Fig. 6:**
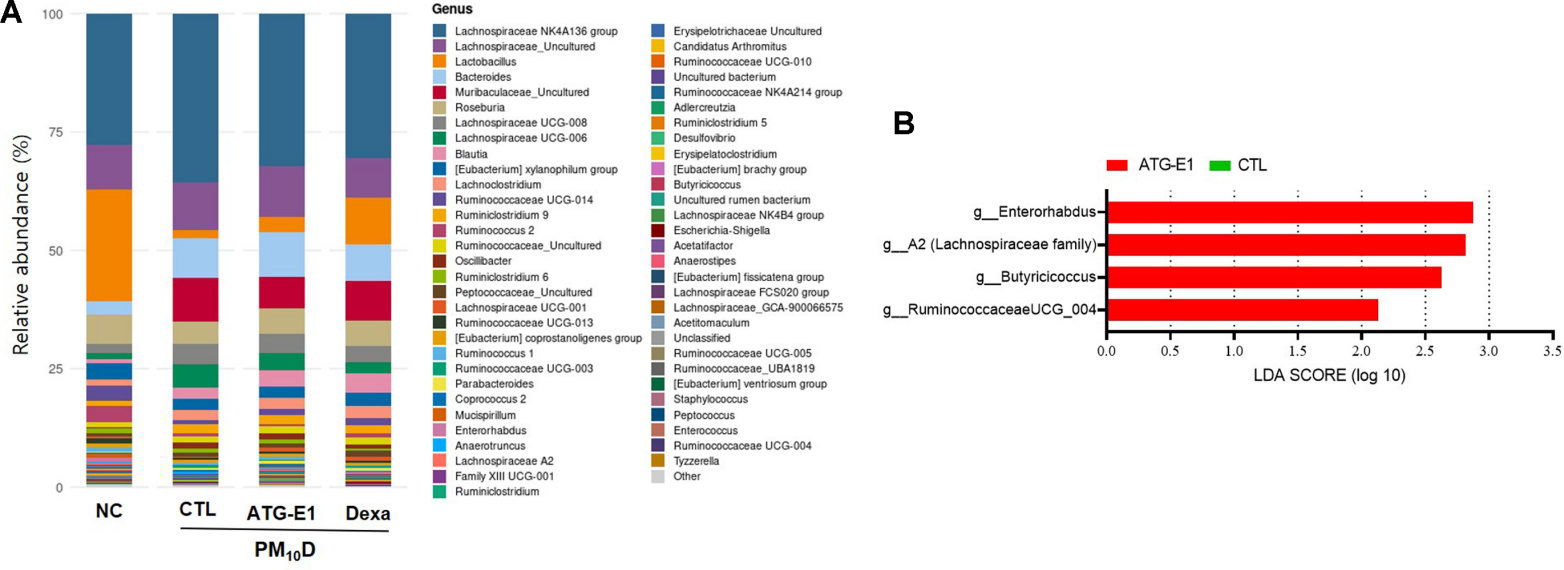
*L. paracasei* ATG-E1 remodels gut microbiome composition in PM_10_D-exposed mice. (**A**) Relative abundance (%) of bacterial communities at the genus level. (**B**) Linear discriminant analysis (LDA) effect size (LEfSe) analysis revealed significant differences in taxa profiles between the control and ATG-E1-treated groups. LDA scores (log10) > 2.0 and adjusted *p* < 0.1 are shown. No taxa were significantly enriched in the CTL group at the applied LDA threshold.

To investigate whether ATG-E1 treatment was associated with changes in gut microbiota composition, we analyzed the fecal microbiota of each group after excluding one low-depth ATG-E1 sample from the 16S rRNA-based microbiome dataset. The most common genus across all groups was *Lachnospiraceae* NK4A136. The *Lactobacillus* genus showed a relative abundance of 23.4% in the NC group but less than 10% in the other groups (Fig. 6A). Notably, despite oral administration of *L. paracasei* ATG-E1, the relative abundance of the *Lactobacillus* genus did not show a marked increase in the ATG-E1-treated group. This observation may be attributable to the limited taxonomic resolution inherent to genus-level 16S rRNA sequencing, which may not reliably distinguish the administered ATG-E1 strain from endogenous *Lactobacillus*-related taxa [26]. In addition, probiotic-mediated effects may occur through transient microbial interactions without stable colonization [27]. To identify differentially enriched taxa between the PM_10_D group and the ATG-E1-treated group, LEfSe analysis was conducted. The LDA score histogram indicated differentially enriched taxa between groups (Fig. 6B). *Enterorhabdus*, A2 (family *Lachnospiraceae*), *Butyricicoccus*, and Ruminococcaceae UCG_004 were enriched in the ATG-E1-treated group, suggesting that ATG-E1 treatment was associated with altered relative abundance of these taxa.

### *L. paracasei* ATG-E1 Treatment Was associated with Altered Fecal Microbial Metabolite Profiles

**Fig. 7:**
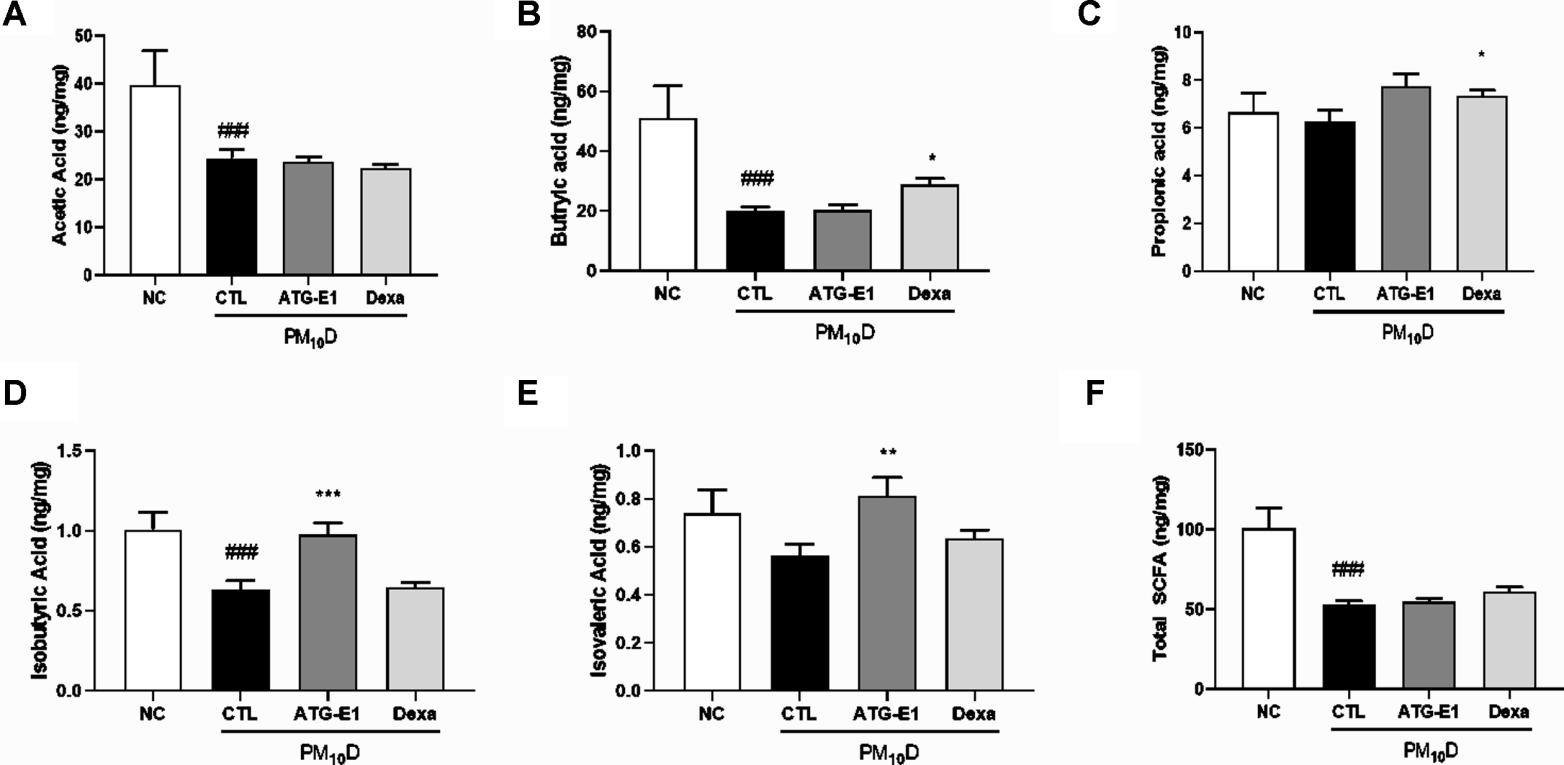
*L. paracasei* ATG-E1 treatment is associated with altered fecal fatty acid profiles, particularly increased BCFAs. Fecal concentrations of individual and total short-chain fatty acids (SCFAs) were determined. Panels (**A**), (**B**), (**C**), (**D**), (**E**) show the levels of acetic acid, butyric acid, propionic acid, isobutyric acid, and isovaleric acid, respectively. (**F**) represents the total SCFA concentration. ATG-E1 treatment was associated with increased fecal isobutyric acid and isovaleric acid levels, whereas major SCFAs showed limited changes compared with the PM_10_D-treated control group. Results are expressed as the mean ± standard error of the mean (SEM). ^###^*p* < 0.001 compared to the normal control (NC) group. *p < 0.05, **p < 0.01, ***p < 0.001 compared to the PM_10_D-treated control (CTL) group.

To investigate whether ATG-E1 treatment was associated with changes in fecal microbial metabolites, we analyzed fecal SCFA and branched- chain fatty acid (BCFA) profiles. PM_10_D exposure reduced fecal acetic acid, butyric acid, and total SCFA levels compared with the NC group (Fig. 7A, 7B and 7F). ATG-E1 treatment was associated with changes in the fecal fatty acid profile, most notably increased levels of the BCFAs isobutyric acid and isovaleric acid (Fig. 7D and 7E). In contrast, major SCFAs, including butyric acid and propionic acid, were not significantly increased in the ATG-E1-treated group compared with the PM_10_D group (Fig. 7B and 7C). Dexamethasone significantly restored butyric and propionic acid levels but had little effect on fecal BCFA levels. These results indicate that ATG-E1 treatment was associated with alterations in fecal microbial metabolite profiles, characterized by increased levels of BCFAs, rather than a broad restoration of major SCFAs.

### *L. paracasei* ATG-E1 Possesses Both Antitussive and Expectorant Activities

**Fig. 8:**
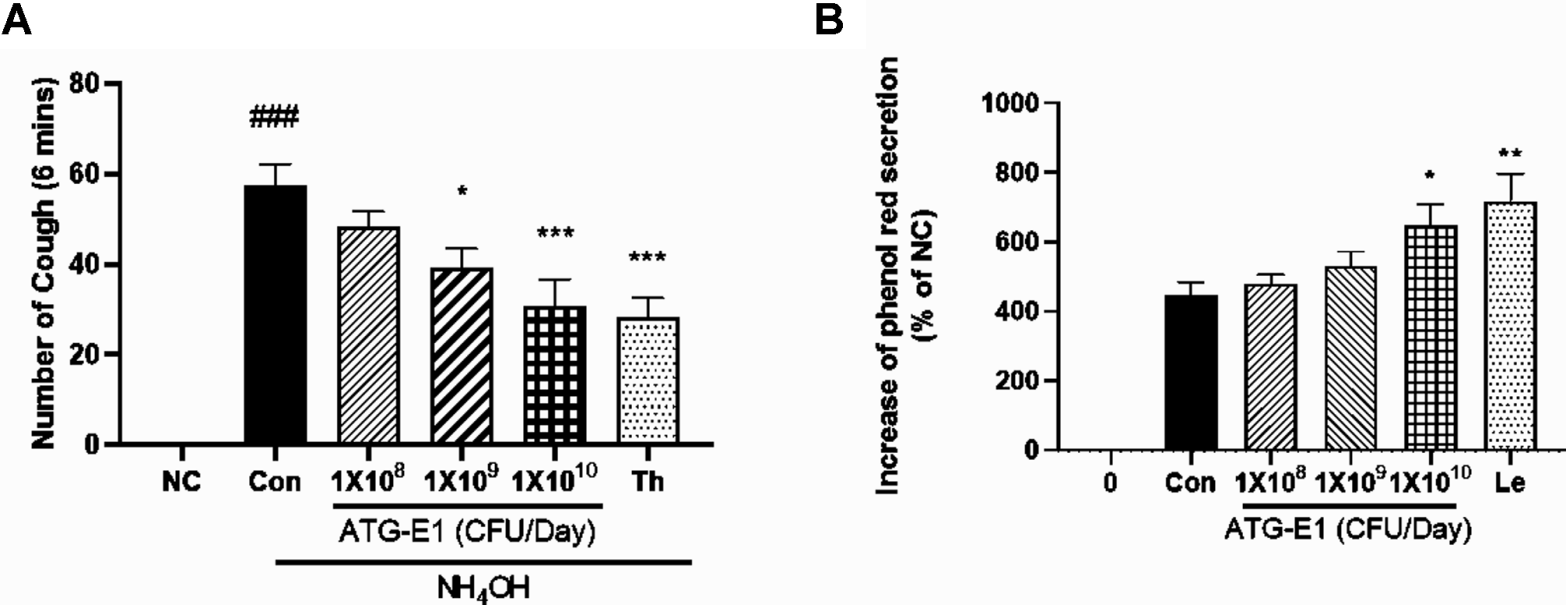
*L. paracasei* ATG-E1 alleviates cough and promotes mucus secretion. (**A**) Antitussive (anti-cough) effect of ATG-E1 was assessed in an ammonia (NH_4_OH)-induced cough model. The number of coughs was counted for 6 min after administering different doses of ATG-E1. Theobromine (Th) was used as a positive control. (**B**) Expectorant effect of ATG-E1 was evaluated by measuring the secretion of phenol red from the trachea. Different doses of ATG-E1 (1 × 10^8^, 1 × 10^9^, 1 × 10^10^ CFU/Day) were administered, with Levodropropizine (Le) used as the positive control. Data are expressed as the percentage increase relative to the normal control (% of NC). Data are presented as the mean ± standard error of the mean (SEM). ^###^*p* < 0.001 compared to the normal control (NC) group. *p < 0.05, **p < 0.01, ***p < 0.001 compared to the vehicle-treated control (Con) group.

Owing to the evidence of its anti-inflammatory activity, we investigated the therapeutic potential of ATG-E1 against respiratory symptoms, specifically cough and mucus hypersecretion. In the ammonia-induced cough model, ATG-E1 treatment significantly suppressed cough frequency compared to the vehicle control, demonstrating a clear antitussive effect (Fig. 8A). To assess its effect on mucus clearance, we measured phenol red secretion. ATG-E1 administration significantly enhanced the phenol red output, indicating an expectorant effect (Fig. 8B). These data demonstrate that ATG-E1 functionally alleviates the major symptoms of airway inflammation.

## Discussion

This study demonstrated that administration of *L. paracasei* ATG-E1 attenuated PM_10_D-induced airway inflammation in a murine model. In contrast to previous analyses focused on prevention [28], the present study evaluated a therapeutic regimen in which ATG-E1 administration was initiated after airway inflammation had been induced. ATG-E1 treatment reduced lung inflammation in association with attenuation of inflammatory signaling responses, improvement of cough- and mucus-related symptoms, and changes in gut microbiota composition and fecal metabolite profiles. Specifically, ATG-E1 attenuated PM_10_D-induced lung inflammation and tissue damage. Histological, BALF, and flow cytometric analyses confirmed a reduction in the number of inflammatory cells in lung tissues (Table 1, Fig. 1B−1E). This reduction in immune cell influx was accompanied by the suppression of pro-inflammatory mediators at both the protein and mRNA levels (Figs. 2 and 3). Notably, the levels of neutrophils, which are key drivers of acute PM-induced lung injury [29-30], were significantly reduced. This reduction was accompanied by a marked decrease in key neutrophil-attracting chemokines, such as CXCL1 and MIP-2. This effect was more pronounced than that of dexamethasone, suggesting that ATG-E1 may have distinct effects on neutrophil-associated inflammatory responses. Furthermore, the attenuation of collagen deposition in the lungs indicates a potential antifibrotic effect. This suggests that ATG-E1 may also help mitigate chronic structural changes associated with airway inflammation (Fig. 1B).

This study also examined inflammatory signaling pathways associated with the therapeutic effects of ATG-E1. In the PM_10_D-induced airway inflammation model, IκBα and ERK phosphorylation were significantly increased, whereas JNK and p38 phosphorylation were not significantly altered in the quantitative analysis. ATG-E1 treatment reduced PM_10_D-induced IκBα and ERK phosphorylation, suggesting attenuation of the inflammatory signaling events activated under these experimental conditions. However, because JNK and p38 were not significantly activated in this model, these data do not establish intrinsic pathway selectivity of ATG-E1. Rather, the findings indicate that ATG-E1 reduced the NF-κB- and ERK-related responses that were elevated following PM_10_D exposure. This reduction may partly explain the observed decrease in inflammatory mediators such as TNF-α and IL-1α, which are regulated by NF-κB- and ERK-associated signaling pathways [31-32].

In addition to modulating signaling pathways, ATG-E1 reduced the expression of markers associated with inflammatory cell death. ATG-E1 treatment markedly suppressed the expression of caspase-1 and IL-1α, which are key mediators of pyroptosis (Fig. 5). Pyroptosis is a lytic form of cell death that releases potent inflammatory molecules, amplifying the inflammatory cascade and causing tissue damage [33-34]. By reducing these markers, ATG-E1 may attenuate inflammatory signaling processes associated with pyroptosis.

Furthermore, our transcriptome analysis provided a broader understanding of the immunomodulation involved. KEGG pathway analysis confirmed that the cytokine-cytokine receptor interaction pathway was significantly suppressed (Supplementary Figs. 1 and 2). This pattern involved the downregulation of multiple immune-related signaling molecules. For instance, the reduced expression of multiple chemokines (CXCL1, CXCL4, and CXCL7) and their receptors (CCR3 and CCR7) suggests that ATG-E1 was associated with the attenuation of immune cell recruitment-related signaling in inflamed lung tissues. Moreover, the downregulation of IL-5RA and CCR3 expression suggests reduced Th2-associated inflammatory signaling [35]. ATG-E1 also reduced the expression of the costimulatory molecule CD40. This reduction may indicate attenuation of adaptive immune activation-related responses. These transcriptomic changes support the observed anti-inflammatory effects of ATG-E1.

From a preclinical perspective, our findings are highly relevant to the pathophysiology of inflammatory airway diseases, such as asthma, chronic obstructive pulmonary disease, and chronic bronchitis. For the expectorant effect, experimental data from the model suggested involvement of a dual-action mechanism. In the PM_10_D-induced inflammatory model, we observed a trend of decreased *Muc5ac* expression (Fig. 3I), suggesting ATG-E1 may normalize pathological mucus hypersecretion by attenuating upstream inflammatory signals. Conversely, in a functional assay, ATG-E1 enhanced phenol red secretion, indicating the expectorant effect (Fig. 8B). Taken together, these findings suggest that ATG-E1 may modulate mucus-related responses rather than simply suppressing or increasing secretion. Furthermore, ATG-E1 may alleviate respiratory symptoms through modulation of sensory irritation-related genes. Our results showed significant suppression of PM_10_D-induced increases in the mRNA levels of *Tarc* and *Trpv1*. TARC, also known as CCL17, attracts Th2 cells and is involved in allergic inflammation. Furthermore, Transient Receptor Potential (TRP) channels (*Trpv1* and *Trpa1*) present on airway sensory nerves mediate neurogenic inflammation and the cough reflex [36-37]. These changes suggest that ATG-E1 may affect both inflammatory and sensory irritation-related responses triggered by PM exposure. These findings further support the potential of ATG-E1 as a therapeutic candidate for air-pollution-induced respiratory inflammation.

In parallel with pulmonary anti-inflammatory effects, ATG-E1 treatment was associated with changes in gut microbiota composition and fecal metabolite profiles. The existence and importance of the gut-lung axis in regulating respiratory health are well established [10, 38-40]. PM_10_D exposure was associated with reduced fecal SCFA levels, including acetic acid, butyric acid, and total SCFAs (Fig. 7A, 7B, 7F). Notably, the most prominent metabolite changes associated with ATG-E1 treatment were increases in the fecal BCFAs such as isobutyric acid and isovaleric acid, whereas the major SCFAs showed only limited changes compared with the PM_10_D group (Fig. 7D and 7E).

This metabolic alteration occurred alongside shifts in gut microbiota composition, including increases in *Enterorhabdus* and *Butyricicoccus* following ATG-E1 treatment. Because BCFAs can be generated through microbial amino acid fermentation, the concomitant increase in fecal BCFAs and decrease in branched-chain amino acids (BCAAs) (Supplementary Fig. S3) is consistent with a possible shift in microbial amino acid metabolism following ATG-E1 treatment [41]. However, the present study did not determine whether ATG-E1 directly produced or modified BCFAs, nor did it include isotope tracing or direct metabolic flux analysis to demonstrate conversion of BCAAs into BCFAs. Therefore, the observed BCFA alterations are more appropriately interpreted as microbiome-associated metabolic changes that may involve altered microbial amino acid fermentation and broader community-level interactions, rather than direct evidence of BCFA production by ATG-E1 or BCFA-mediated anti-inflammatory effects.

Concurrently, the increase in *Butyricicoccus*, a well-documented butyrate-producing bacterium, was observed [42-43]. However, this enrichment did not correspond to a significant increase in fecal butyric acid levels in the ATG-E1-treated group compared with the PM_10_D group (Fig. 7B). This discrepancy may reflect complex factors, such as substrate availability, rapid colonic absorption of butyrate, or altered metabolic cross-feeding pathways. Therefore, increased fecal BCFAs should be interpreted as a microbiome-associated metabolic signature of ATG-E1 treatment rather than direct evidence of an anti-inflammatory mechanism. Although BCFAs have been implicated in host immune regulation, they are primarily generated through microbial amino acid fermentation and may also reflect increased proteolytic activity in the gut. Thus, their biological effects may depend on the intestinal context, metabolite concentration, microbial ecosystem, and host inflammatory status. Importantly, the present study did not determine whether fecal BCFAs entered the systemic circulation or lung tissue. Accordingly, the observed increase in fecal BCFAs alone does not establish that these metabolites mediated systemic or pulmonary anti-inflammatory effects. The reduction in PM_10_D-induced T cell accumulation and activation in Peyer’s patches (Supplementary Fig. S4) suggests that ATG-E1 may influence gut-associated immune responses; however, further studies are required to determine whether these changes are causally linked to reduced lung inflammation.

This study has several limitations. Murine models were used in all experiments; therefore, the results should be confirmed in human clinical studies. In addition, although ATG-E1 treatment was associated with changes in gut microbiota composition, fecal metabolite profiles, and reduced airway inflammation, the present study does not establish a causal gut–lung axis mechanism. Gut-derived metabolites were not measured in serum, plasma, or lung tissue, and causal intervention experiments, such as fecal microbiota transplantation, antibiotic-mediated microbiota depletion, germ-free mouse experiments, or metabolite supplementation, were not performed. Therefore, further studies are needed to determine whether gut microbiota- or fecal metabolite-associated changes directly contribute to the pulmonary anti-inflammatory effects of ATG-E1. In addition, although increased fecal BCFAs and decreased BCAAs are consistent with altered microbial amino acid metabolism, the present study did not directly demonstrate BCAA-to-BCFA conversion or establish BCFAs as causal anti-inflammatory mediators.

In conclusion, *L. paracasei* ATG-E1 alleviated PM_10_D-induced airway inflammation and respiratory symptoms in mice. These effects were associated with reduced inflammatory cell infiltration, decreased inflammatory mediator production, attenuation of PM_10_D-activated IκBα and ERK phosphorylation, and reduced expression of caspase-1 and IL-1α in lung tissue. ATG-E1 treatment was also associated with changes in gut microbiota composition and fecal metabolite profiles, including increased fecal BCFA levels. However, further studies are required to determine whether gut microbiota- or fecal metabolite-associated changes directly contribute to pulmonary immunomodulation. These findings support ATG-E1 as a promising microbiome-associated therapeutic candidate for PM_10_D-induced airway inflammation.

## Supplemental Materials

Supplementary data for this paper are available on-line only at http://jmb.or.kr.



## Figures and Tables

**Table 1:** Modulation of immune cell profiles in the BALF and lungs by *L. paracasei* ATG-E1 in a model of airway inflammation.