# Supplementary material for: Lacticaseibacillus paracasei ATG-E1 Alleviates Particulate Matter-Induced Airway Inflammation in Association with Altered Inflammatory Signaling, Gut Microbiota, and Fecal Metabolites
Source: J Microbiol Biotechnol. 2026 Aug 6;36:e2604029. doi: 10.4014/jmb.2604.04029 (PMC13454835; doi:10.4014/jmb.2604.04029)
Supplement: Supplementary file 1 [file jmb-36-e2604029-supple.pdf]

Supplementary Fig S1

J. Microbiol. Biotechnol. 2026. 36: e2604029
https://doi.org/10.4014/jmb.2604.04029

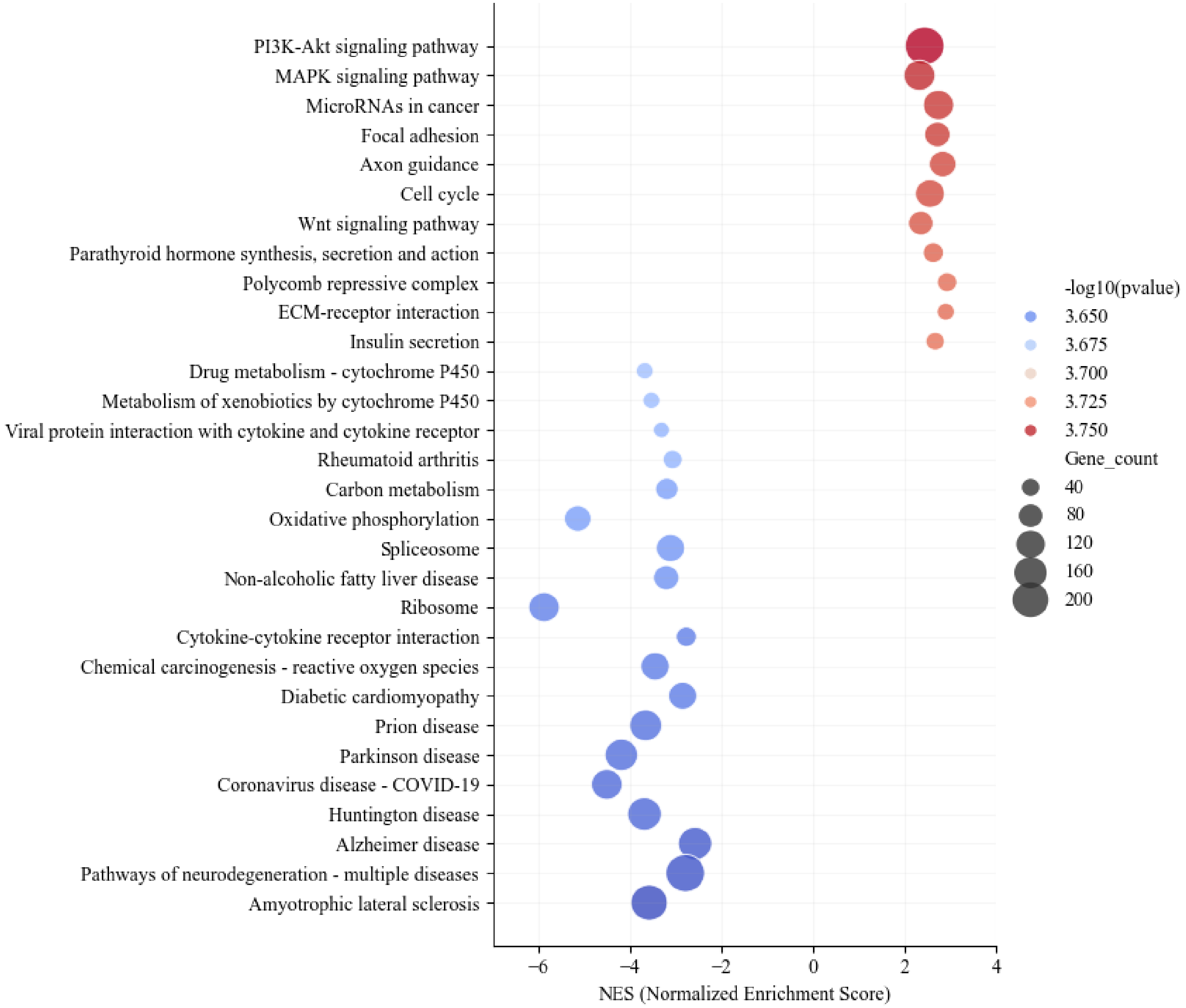

Supplementary Fig S2

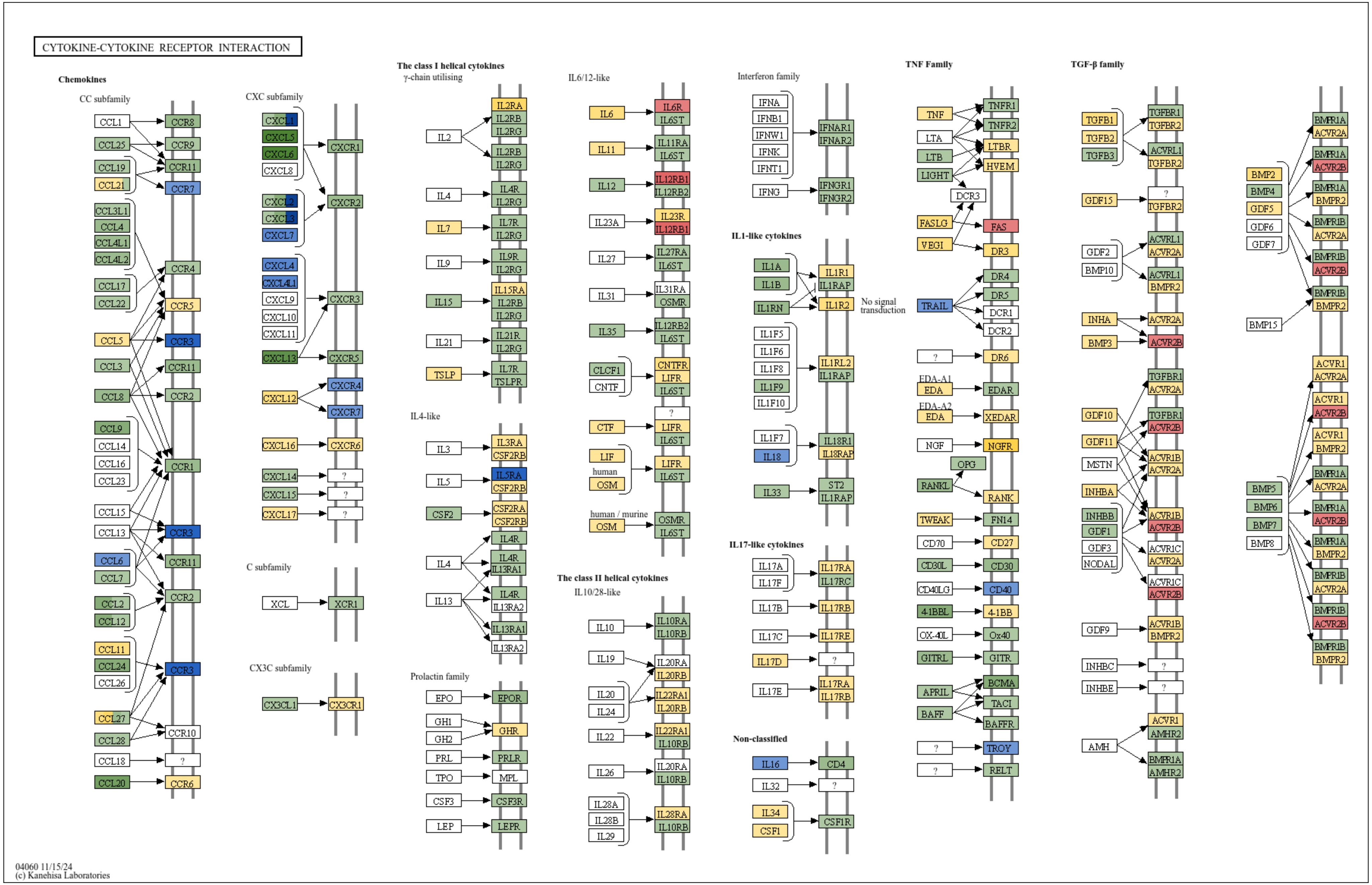

Supplementary Fig S3

Amino acids (mg/mg cecal)	NC	CTL	*L. paracasei* ATG-E1	Dexa
Alanine	858.6±49.1	109.5±32.7$^{\#\#}$	111.80±100.1	944.7±12.4
Arginine	109.3±18.0	182.8±20.0	91.9±27.0*	141.7±23.9
Asparagine	155.7±7.5	186.1±7.7	152.9±20.5	186.0±7.1
Aspartic acid	331.2±13.2	308.9±9.1	313.6±32.4	361.0±13.8
Cysteine	98.3±15.4	84.1±15.5	102.6±9.2	114.0±9.5
Glutamine	2341.0±95.2	2957.8±340.9	2099.3±228.3	2680.3±90.0
Glutamic acid	1112.5±40.1	1301.6±14.3	1279.8±102.7	1147.5±30.1
Glycine	170.0±20.0	221.2±20.7	268.3±37.5	260.6±28.6
Histidine	36.4±2.1	49.5±2.4	38.3±5.6	47.6±1.9
Isoleucine	378.2±15.0	534.9±14.7$^{\#\#}$	398.4±4.1***	431.6±9.4**
Leucine	756.8±34.6	975.1±26.4$^{\#\#}$	795.7±82.7*	889.8±19.4
Lysine	648.2±33.0	841.2±49.5	792.7±90.9	814.3±19.5
Methionine	234.5±11.4	398.2±36.7$^{\#\#\#}$	261.6±29.6**	285.6±7.1*
Phenylalanine	379.8±15.1	483.7±9.4$^{\#\#}$	411.3±37.3*	441.6±6.9
Proline	309.4±12.9	373.5±9.9	392.4±38.1	350.9±10.8
Serine	206.7±10.6	178.0±14.2	202.1±21.8	254.5±12.6*
Threonine	309.5±11.3	444.5±10.3$^{\#\#\#}$	347.9±34.4**	383.7±14.9
Tryptophan	41.0±3.1	90.6±9.4$^{\#\#\#}$	50.3±10.3**	57.7±2.4*
Tyrosine	333.5±11.9	458.6±11.2$^{\#\#\#}$	365.7±34.6**	387.6±8.6*
Valine	454.7±18.3	599.8±11.0$^{\#\#\#}$	510.3±49.6	521.0±13.2

NC: BALB/c normal mice; CTL: PM_{10}D-sensitized control mice; Dexa: 3 mg/kg dexamethasone-treated PM_{10}D-sensitized mice; *L. paracasei* ATG-E1 : *L. paracasei* ATG-E1 treated PM_{10}D-sensitized mice. Data are expressed as the mean ± SEM (n = 8). $^{\#\#}p < 0.01$ and $^{\#\#\#}p < 0.005$ vs NC; $^{*}p < 0.05$, $^{**}p < 0.01$ and $^{***}p < 0.005$ vs CTL.

Supplementary Fig S4

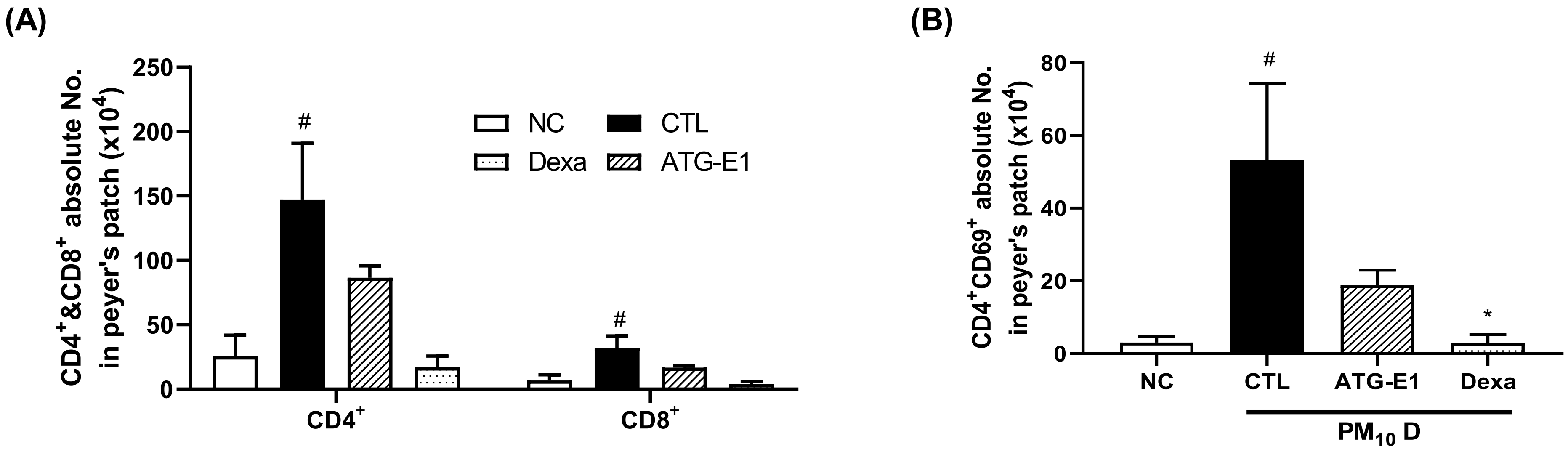

Supplement Fig. S5. Rarefaction curves demonstrating sequencing depth saturation across fecal microbiome samples.

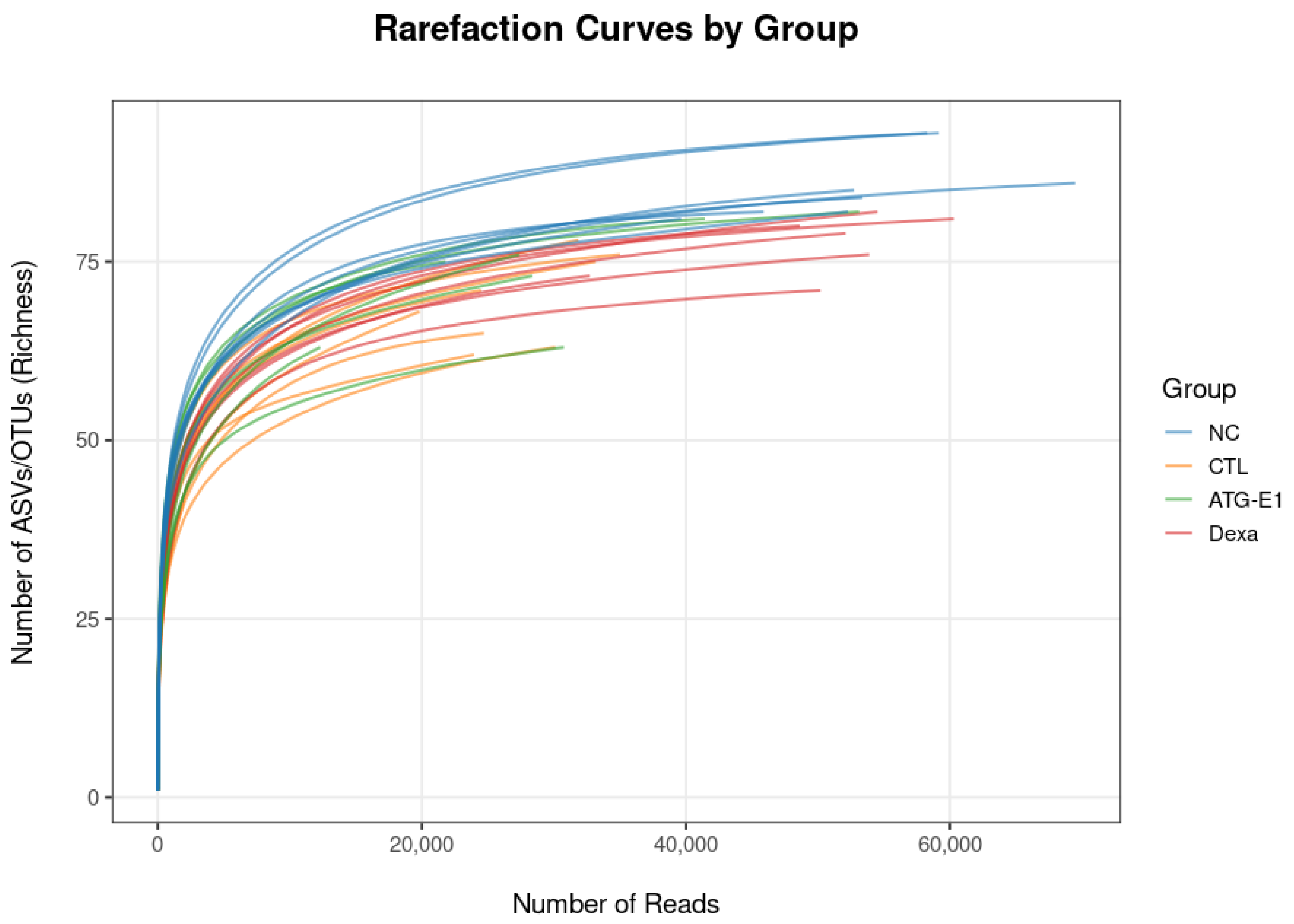

Supplementary Table S1. Sequencing depth and richness metrics for all microbiome samples.

Sample ID	Group	Total Reads	Observed Richness
NC1	NC	58268	93
NC2	NC	45884	82
NC3	NC	69485	86
NC4	NC	59144	93
NC5	NC	39634	81
NC6	NC	52727	85
NC7	NC	52273	82
NC8	NC	53367	84
CTL1	CTL	35019	76
CTL2	CTL	31850	78
CTL3	CTL	24707	65
CTL4	CTL	33145	75
CTL5	CTL	19824	68
CTL6	CTL	30113	63
CTL7	CTL	24490	71
CTL8	CTL	23955	62
LH1	ATG-E1	53115	82
LH2	ATG-E1	41424	81
LH3	ATG-E1	28377	73
LH4	ATG-E1	21796	75
LH5	ATG-E1	30760	63
LH6	ATG-E1	12303	63
LH7	ATG-E1	27383	76
LH8[1]	ATG-E1	4576	67
Dexa1	Dexa	60291	81
Dexa3	Dexa	48616	80
Dexa4	Dexa	52097	79
Dexa5	Dexa	50191	71
Dexa6	Dexa	32723	73
Dexa7	Dexa	54515	82
Dexa8	Dexa	53881	76

[1]LH8 yielded only 4,576 reads and was excluded from 16S rRNA-based microbiome analyses. After excluding LH8, diversity analyses were performed at a rarefaction depth of 12,000 reads per sample.
